# Which one is better, Wallace or Bricker?

**Published:** 2007

**Authors:** Naval Khurana, Aneesh Srivastava

**Affiliations:** Department of Urology and Renal Transplant, SGPGIMS, Lucknow, India. E-mail: drrgoyal@yahoo.com

## SUMMARY

This is a retrospective study in 198 patients who underwent radical cystectomy in carcinoma urinary bladder from July 1997 to July 2003. Eighty-six patients underwent Bricker ureterointestinal anastomosis (UIA) and 112 underwent Wallace UIA. Bricker UIA was considered two anastomotic units while Wallace UIA was considered a single unit. Therefore, there were 162 (59%) total Bricker UIA compared to 112 (41%) Wallace UIA. There were three strictures (1.85%) in the Bricker group and five strictures (4.46%) in the Wallace group (statistically insignificant). A total of 33 patients (17%) in the study received neoadjuvant radiation, salvage radiation or prior radiation. Of the 33 radiated cases (48 anastamosis) there were three strictures. Although the stricture rate between radiated (6.25%) and nonradiated (2.21%) anastomosis was considerable, this was not statistically significant. There was no statistically significant difference between stricture rates in small bowel vs. large bowel diversions or in the groups comparing 5Fr feeding tubes to 7Fr single J stents.

## COMMENTS

The choice between a Bricker anastomosis and Wallace anastamosis is mostly based on surgeon preference. Criticism of Wallace anastamosis is due to the rare recurrence at the UIA or the possibility of a stone which may obstruct both systems. A recurrence at this junction may affect both collecting systems and can cause bilateral ureteral obstruction and subsequent renal failure. Also, the subsequent need for a nephroureterectomy involving a Wallace anastomosis can be complex and requires reimplanting in a Bricker fashion. Bricker anastomosis, on the other hand, has been criticized on the basis of an increased risk of stricture formation and increased operative time required, despite any published evidence to support these claims.

Although commonly performed, these procedures have not been properly studied in randomized fashion, except a few small observational studies.[1,2] The limitations of this study[3] are retrospectivity, selection bias-each surgeon had his or her preferred technique (several different surgeons performed the Bricker anastomosis while the majority of the Wallace anastomosis were performed by a single surgeon) and mean follow-up less than two years, likely suboptimal for a study of this nature and the possibility that the study may have been underpowered to establish a clinically relevant difference. In spite of all this there was no statistically significant difference between the stricture rates in these two groups. This in turn suggests that the final decision about the technique of UIA should remain within the realm of surgeon preference. The authors are to be commended for presenting important data on this issue, which has puzzled the surgeons for the last few generations. The overall stricture rate was 2.9%, in line with stricture rates from other series in the literature.[4] There was a trend for higher stricture rate in previously radiated cases (6.25% vs. 2.21%). It seems likely this finding would have reached statistical significance with a larger sample size. The early mean time for appearance of stricture (8.8 months) reflects that most are due to ureteral ischemia. The finding that only 25% of strictures responded to endoscopic treatment is similar to other reports. We should remember that a stricture of a Wallace anastomosis can affect both renal units, as it did in three of five cases in this series and, thus, can have far more devastating effects. Additional data will be required to address these concerns. One hopes that this article will stimulate further work about this important aspect of urinary diversion. One final comment is not to conclude from uncontrolled series that the risks associated with ureteroenteric stricture are equivalent among various types of diversion and bowel segments. Prospective, randomized studies would be the preferred but are unlikely to be performed, as surgeons typically have strong preferences on the type of anastomosis performed, segment of bowel used and type of stent or whether a stent is used.
